# CARMA II: A ground vehicle for autonomous surveying of alpha, beta and gamma radiation

**DOI:** 10.3389/frobt.2023.1137750

**Published:** 2023-03-31

**Authors:** Bahman Nouri Rahmat Abadi, Andrew West, Harriet Peel, Matthew Nancekievill, Christopher Ballard, Barry Lennox, Ognjen Marjanovic, Keir Groves

**Affiliations:** ^1^ Manchester Centre for Robotics and AI, University of Manchester, Manchester, United Kingdom; ^2^ Sellafield Ltd., Carlisle, United Kingdom

**Keywords:** autonomous ground vehicle, ROS, radiation detection, costmap, state machine, experimental

## Abstract

Surveying active nuclear facilities for spread of alpha and beta contamination is currently performed by human operators. However, a skills gap of qualified workers is emerging and is set to worsen in the near future due to under recruitment, retirement and increased demand. This paper presents an autonomous ground vehicle that can survey nuclear facilities for alpha, beta and gamma radiation and generate radiation heatmaps. New methods for preventing the robot from spreading radioactive contamination using a state-machine and radiation costmaps are introduced. This is the first robot that can detect alpha and beta contamination and autonomously re-plan around the contamination without the wheels passing over the contaminated area. Radiation avoidance functionality is proven experimentally to reduce alpha and beta contamination spread as well as gamma radiation dose to the robot. The robot’s survey area is defined using a custom designed, graphically controlled area coverage planner. It was concluded that the robot is highly suited to certain monotonous room scale radiation surveying tasks and therefore provides the opportunity for financial savings, to mitigate a future skills gap, and provision of radiation surveys that are more granular, accurate and repeatable than those currently performed by human operators.

## 1 Introduction

Recent global concerns over energy cost and security of supply have refocused interest in nuclear energy as a reliable, low carbon energy source, that can often be domestically generated (HM Government, 2022). In the United Kingdom, new large-scale nuclear power reactors are being constructed and there are plans for a fleet of Small Modular Reactors (SMRs) to come online in the 2030s (HM Government, 2020). In addition to the construction of new nuclear facilities, the United Kingdom has many legacy facilities that no longer produce power and are going through post operational clean out and decommissioning. Sellafield Ltd’s site in West Cumbria is the largest and most complex of these facilities by far and has been home to several generations of nuclear reactors; some of the early reactors were built to produce materials for nuclear weapons (1940s) and later reactors (from 1956) produced power for businesses and homes (HM Government, 2019).

One of the key concerns surrounding both the construction of new nuclear facilities and decommissioning old facilities is financial cost. Current estimates for completing the new large scale nuclear power plant, Hinkley Point C, which is under construction in the United Kingdom, stands at £25–26 billion and is planned to start producing energy in 2027 (Financial-Times, 2022). Meanwhile, Sellafield currently costs the United Kingdom government over £2.3 billion per year and the most recent estimate from the Nuclear Decommissioning Authority (NDA) is that decommissioning of Sellafield will take 100 years and cost approximately £110 billion in total (un-discounted) (Nuclear Decommissioning Authority, 2022). Something that is common to both Nuclear decommissioning and the building of new nuclear power plants in the United Kingdom is that, cost and timescale of projects generally increase as the projects progress. For example, the first estimate for the cost of Hinkley Point C given in 2016 was £18 billion (now risen to 25–26 billion) with a date for starting generating power of 2025 (now delayed to 2027) (BBC-News, 2017).

One of the bottlenecks that can lead to time and cost overruns is the availability of suitably trained radiation safety experts, termed health physics surveyors or monitors (HM Government, 2015). In both decommissioning and building of new nuclear facilities, there are a large number of tasks that require clearance from health physics personnel, such as routine inspection activities and moving of personnel and material. Health physics surveyors are in fact required, usually by law or regulation (Health and Safety Executive, 2018), during all phases of a nuclear facilitie’s life, i.e., construction, operation and decommissioning (Tsitsimpelis et al., 2019). Poor availability of health physics monitoring can affect scheduling of work and cause bottlenecks, especially if surveyors are also performing reactive work.

Currently, within the nuclear industry, there is a shortage of qualified health physics personnel and this is only set to worsen in the coming years. It is estimated that 50 percent of the existing health physics personnel will retire in the next 10–15 years, and this is not presently matched by recruitment and training of new staff. This is compounded by the predicted increase in demand for health physics monitoring across the nuclear sector (Bryant, 2021).

### 1.1 Using robots to reduce costs and improve radiation surveys

A potential solution to the problem of health physics resourcing, and a way to reduce costs, is the use of robotic systems to perform basic, repetitive health physics surveying tasks, therefore freeing up valuable human specialist time to focus on more sophisticated tasks, such as interpreting data or surveying more complex areas. A significant proportion of the work of a health physics surveyor is dull and repetitive, for example, holding a probe at a fixed distance from a flat surface and moving it at a fixed velocity until an area has been fully covered by the probe; such tasks are well suited to automation. Robotic systems are currently being developed, which are capable of performing such tasks autonomously. Because the robots have localisation and mapping systems, they are able to provide accurate location tagged radiation measurements in the form of datasets and heatmaps. This capability means that accuracy and repeatability of measurements can be improved compared to a human operator. In addition, surveys can be performed more frequently and meticulously due to the reduced need for health physics surveyor resource. Developing and using such robotic systems also significantly improves human safety by providing an interface between the workforce and the work face, where there is potential for exposure to higher than background levels of radiation.

### 1.2 Literature review

Mobile robots in a variety of forms and levels of autonomy are commonly used within nuclear facilities. These robots have been developed and deployed for a variety of purposes, such as decommissioning (Marturi et al., 2017; Marques et al., 2021; Monk et al., 2021; West et al., 2021) and monitoring (Li et al., 2018; Groves et al., 2019; Anderson et al., 2020; Connor et al., 2020; White et al., 2020, 2021; Abd Rahman et al., 2022a). A number of these systems have been trialled and evaluated in active nuclear facilities such as the Fukushima Daiichi nuclear power plant in Japan (Nagatani et al., 2013; Nancekievill et al., 2018) and the Dounreay site in the United Kingdom (Bird et al., 2022; Nancekievill et al., 2023). This research is focused specifically on autonomous ground based robots for radiation monitoring and the remainder of this section provides a brief review of the relevant literature.

The earliest research into the use of autonomous ground vehicles for radiation mapping found by the authors was by Cortez et al. (2008). This work focused on detecting gamma rays and locating point sources. However, at the time of that work mobile robot autonomy was not as well developed as it is today and the robot required prior knowledge about the physical environment and could not avoid dynamic obstacles.

Since the work of Cortez et al. (2008), there has been a breadth of work in the area and the most relevant research found by the authors has been summarised in Table 1. A large fraction of these publications (Hosmar et al., 2017; McDougall et al., 2018; Huo et al., 2020; Kishimoto et al., 2021) focus on using gamma radiation information, which was collected by an autonomous ground vehicle, as an input to solve the inverse problem of localising gamma point sources. For instance, Hosmar et al. (2017) implemented a fully autonomous robot, capable of localising a gamma source. This robot did not require prior knowledge of the environment and was experimentally validated using a two Cs-137 sources with different intensities. Although solving this inverse problem is an interesting research area, it is not the focus of the research reported in this paper. This research is focused on performing autonomous radiation surveys of alpha, beta and gamma sources while reducing radiation dose to the robot as well as contamination spread.

**TABLE 1:** Overview of research work concerning the use of autonomous ground vehicles to survey for radiation.

References	Physical mapping	Static obstacle avoidance	Dynamic obstacle avoidance	Gamma mapping	Localising gamma point sources	Gamma avoidance	Alpha/beta mapping	Alpha/beta avoidance
Cortez et al. (2008)	No	No	No	Yes	No	No	No	No
Zakaria et al. (2017)	No	No	No	Yes	No	No	No	No
Kim et al. (2017)	Yes	Yes	Yes	Yes	No	No	No	No
Hosmar et al. (2017)	No	No	No	Yes	Yes	No	No	No
McDougall et al. (2018)	Yes	Yes	Yes	Yes	Yes	No	No	No
Bird et al. (2018)	Yes	Yes	Yes	No	No	No	Yes	No
Huo et al. (2020)	Yes	No	No	Yes	Yes	No	No	No
Abd Rahman et al. (2020)	Yes	Yes	Yes	Yes	No	No	No	No
Kishimoto et al. (2021)	No	No	No	Yes	Yes	Yes	No	No
Groves et al. (2021)	Yes	Yes	Yes	Yes	No	Yes	No	No
West et al. (2022)	Yes	Yes	Yes	Yes	No	Yes	No	No
Abd Rahman et al. (2022b)	Yes	Yes	Yes	Yes	No	No	No	No
Liu et al. (2022)	Yes	Yes	Yes	No	No	No	Yes	Yes
CARMA II	Yes	Yes	Yes	Yes	No	Yes	Yes	Yes

Other research works have focused on constructing a map of gamma radiation within an environment while minimising the radiation dose received by the robot (Groves et al., 2021; West et al., 2022). Including radiation information within the navigation system’s costmaps to give the robot the ability to avoid regions with high radiation was first developed by Groves et al. (2021) and was later proven to reduce the dose that the robot received by West et al. (2022). This approach works by introducing additional path planning cost to traverse areas with elevated gamma radiation dose rate. In the present work, this costmap approach to minimising the gamma radiation dose to the robot is adopted. However, because the focus is on environments where health physics monitoring is required, high levels of gamma radiation are not expected so gamma avoidance functionality would only impact navigation in unexpected circumstances.

The first autonomous ground vehicle that was designed specifically with health physics surveying in mind was the first version of CARMA (continuous autonomous radiation-monitoring assistant) (Bird et al., 2018). This robotic system was equipped with a ThermoFisher Scientific DP6 probe to survey the environment for alpha and beta radioactive contamination and a ThermoFisher Scientific RadEye G-10 personal dosimeter for measuring gamma radiation. The robot was able to survey unknown environments for alpha, beta and gamma radiation and generate a radiation heatmap of the environment. Although the robot had rudimentary alpha radiation avoidance capabilities, the robot’s mechanical design prevented it from being able to detect alpha contamination before the robot had driven over the contaminated area, which made the radiation avoidance system unsuitable for use in practical situations; this shortcoming was highlighted by the authors at the time. Contamination spread due to fine particulate powders similar to those in nuclear environments can be readily transported by wheels, tracks or feet, being very difficult to clean completely from robot parts, thereby rendering the platform itself partially contaminated (Bird et al., 2022).

The focus of the current research and the reason for developing CARMA II is to address the shortcomings of the first version of CARMA and provide the research required to produce a robot that can relieve the load on health physics personnel both now and in the future. The final column in Table 1 shows the functionality of CARMA II so that it can be directly compared to its predecessors.

## 2 Contributions

In this section the contributions made by this work compared to the state of the art are listed. First, the main research contributions are listed and second, functional improvements to the system are presented.

### 2.1 Research contributions

• The development and practical demonstration of a mobile robot that can detect alpha and beta contamination and autonomously reverse and then navigate around the contamination patch without its wheels becoming contaminated.
• A state machine that controls the robot’s behaviour at a high level and facilitates a range of new functionality, including integration of the waypoint planner alpha and beta contamination avoidance.
• The combination of alpha, beta and gamma radiation as well as obstacles into a single costmap that can be used by the robot’s navigation system.

### 2.2 Functional improvements

• The development of a coverage based waypoint planner that is integrated into the navigation system and allows high level of control by the user, *via* a graphical interface.
• Front and rear dynamic obstacle avoidance using two depth cameras.
• A full hardware redesign including a control base-station and variable height probe for detecting alpha and beta radiation.

## 3 Hardware architecture

**FIGURE 1:**
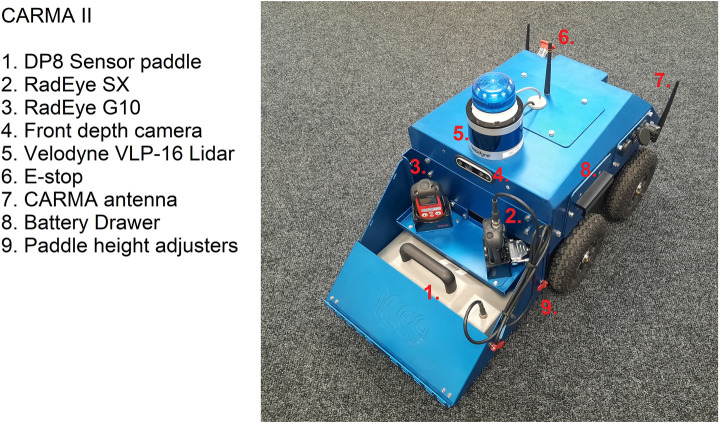
The hardware that has been developed for the CARMA II platform at the University of Manchester.

CARMA II was developed in the Robotics for Extreme Environments Laboratory at The University of Manchester and is depicted in Figure 1. The CARMA II robotic platform presented in this research is a modified Clearpath Jackal (Clearpath-Robotics, 2015). Its hardware has been designed to make it suitable for autonomous monitoring and characterisation of indoor, nuclear facilities. Equipped with a ThermoFisher Scientific RadEye G-10 personal dosimeter and ThermoFisher Scientific RadEye SX connected to a DP8 probe, it can detect alpha, beta, and gamma radiation. Since alpha and beta radiation have low permeability in air, the DP8 probe is mounted low and its height is adjustable. The DP8 probe is mounted to the front of the robot so that alpha and beta contamination can be detected before the robot’s wheels come into contact with radioactive contamination and potentially spread it. CARMA II’s on-board navigation sensor package consists of a Velodyne VLP 16 LiDAR, two Intel Realsense D435i depth cameras, four wheel encoders, and an Inertial Measurement Unit (IMU). The CARMA II Li-Ion battery pack is placed in a convenient drawer to enable the battery to be removed, replaced, and charged with ease. Table 2 summarises this information and provides some additional specifications of the robot.

**TABLE 2:** CARMA II hardware specifications.

Base platform	Jackal (Clearpath)
Alpha/Beta sensors	One ThermoFisher Scientific RadEye SX connected to a DP8
Gamma sensor	One ThermoFisher Scientific RadEye G-10
External navigational sensors	One Velodyne VLP 16 LiDAR, Two intel realsense d435 depth cameras
Internal navigational sensors	Four wheel encoders and one IMU
Communication interface	Encrypted Wi-Fi
External dimensions	450 mm × 500 mm x 400 mm
Weight	25 kg
Run-time	Up to 4 h
Velocity	Up to 1.0 m/s

**FIGURE 2:**
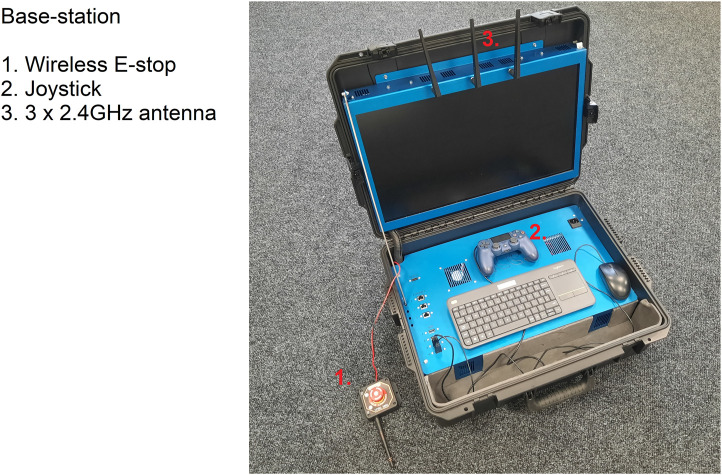
The base-station developed to control the CARMA II platform.

To control CARMA II, a base-station was designed and built as depicted in Figure 2. The base-station consists of a 24” 4K display, a 2.4 GHz Wi-Fi router with three antenna, an embedded PC, a wireless emergency stop and a joystick for manual driving. The CARMA II robotic platform and the base-station communicate wirelessly *via* the router in the base-station. Wireless communications are used for sending commands from the base-station to the platform and for transmitting data from the CARMA II platform to the base-station, prior to it being displayed on the screen.

## 4 Software

**FIGURE 3:**
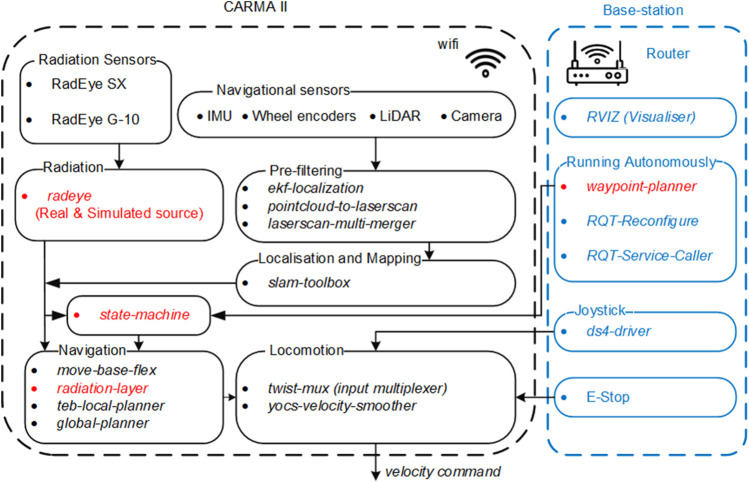
The ROS packages used on CARMA II and the base-station (packages in red text were developed for CARMA II).

The software controlling CARMA II was developed using the robot operating system (ROS) framework. The software is arranged into several ROS packages, some of which are standard packages available in ROS and some are custom packages developed by researchers at the University of Manchester. The robot can be run in manual or autonomous mode and the required packages for either case are depicted in Figure 3. The highlighted packages in red were developed at the University of Manchester.

In manual mode, CARMA II is driven using a joystick controller as shown in Figure 2. Manual mode is generally used for moving the robot around for convenience, or to generate the map that is used by the on-board autonomous algorithms. In both autonomous and manual modes, the physical map is generated by *slam-toolbox* ^1^.

In autonomous mode, the area to be scanned by the robot, herein termed the coverage area, can be selected by an operator at the base-station, using the *waypoint-planner* package, developed in this research. To move the robot autonomously between waypoints generated by the *waypoint-planner*, the standard ROS package *move-base-flex* ^2^ was used. However, additional costmap layers were added to the costmap produced by *move-base-flex* using the *radeye* and *radiation-layer* packages (West et al., 2022). Parameters related to standard and custom packages can be configured using the *dynamic-reconfigure* ^3^ package. For example, the user can set parameters for adjusting the shape of the coverage area and the threshold at which the robot avoids contamination.

In what follows, details regarding the packages used for navigation, radiation detection, and selecting coverage area are provided.

### 4.1 Navigation sensors

The 3D point cloud data from the two depth cameras is converted into 2D laser scans, using the *pointcloud-to-laserscan* ^4^ package. The *velodyne-pointcloud* ^5^ package publishes laser scan data, which is combined with the laser scans derived from the depth cameras, using the *laser-multi-merger* node from the *ira-laser-tools* ^6^ package. The combined laserscan message is then delivered to the SLAM algorithm alongside data from the IMU and Wheel encoders.

### 4.2 Integrating information from radiation sensors

**FIGURE 4:**
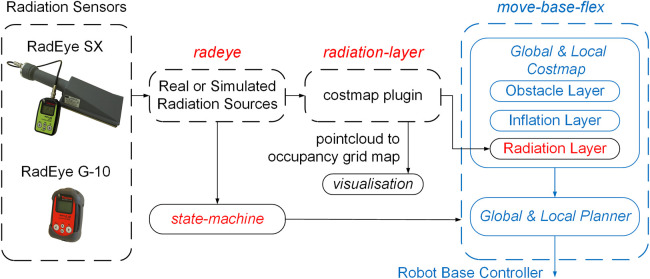
The schematic model of the sensors and packages for the radiation-aware navigation and mapping.

The schematic model of the radiation detectors and the related packages for radiometric mapping are shown in Figure 4. The *radeye* ^7^ package, which is a driver for the RadEye G-10 and RadEye SX and was developed as part of this research and publishes data from the radiation sensors using a standard ROS message^8^ (West and Smith, 2021) which contains the radiometric information and time of observation.

The *radiation-layer* ^9^ package adds data from the radiation sensors to the costmap that is used by the navigation system. The level of risk to the robot from measured radiation is expressed in a costmap so that CARMA II can avoid radiation in the same way in which it avoids obstacles. Different parameters such as radiation thresholds and inflation radius can be tuned by the user for better performance in radiation informed navigation. The concept of the layered costmap with radiation layers is detailed in our previous work (West et al., 2022) and is not a contribution of this paper. In the case of CARMA II, the modified *radiation-layer* package takes into account alpha, beta and gamma radiation and integrates these measurements into the relevant costmaps.

### 4.3 Packages for navigation

#### 4.3.1 Waypoint planning

To allow custom shaped areas to be autonomously monitored the *waypoint-planner* ^10^ package was written to generate a path of concentric squares that are definable by the user through a series of drag and drop interactive markers. The number of markers and inner loops can be selected using *dynamic-reconfigure* package. Using this method customised areas can be selected for monitoring.

**FIGURE 5:**
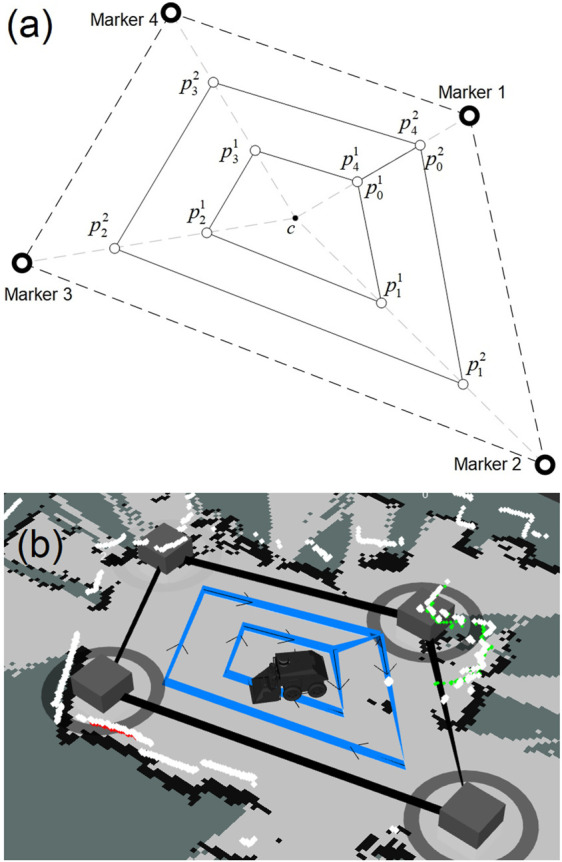
**(A)**: Waypoint planner schematic diagram with four markers and two loops, $p_{i}^{j}$ : The *i*th waypoint in the *j*th loop, *c*: The geometric center of the interactive markers, **(B)**: Screenshot of the waypoint planner with four markers and two loops in the visualiser; the user movable markers are the grey boxes and the waypoint planner’s path is the blue line.

In Figure 5, the *waypoint-planner* with four interactive markers and two inner loops is demonstrated. Figure 5A is a schematic representation while Figure 5B is a screenshot from the graphical interface. To determine the position of the waypoints, the geometric center of the interactive markers, denoted as *c*, is first calculated by taking the arithmetic mean of the marker’s position in the *x* and *y* directions. Radial lines are then drawn from point *c* to each interactive marker. Considering the number of loops, the position of waypoints in each radial line distributed equally between point *c* and the corresponding interactive marker is obtained and the concentric loops are plotted as depicted in Figure 5A.

Once all waypoints are determined, they are formed into a list. In the *waypoint-planner* algorithm, each waypoint is denoted by $p_{i}^{j}$ , in which *i* indicates the waypoint number in the *j*th loop, referred to as *L*_*j*_. The first point in the list is $p_{0}^{1}$ the subsequent waypoints in the list are formed by moving clockwise around the inner loop until the final point in the loop, $p_{4}^{1}$ in Figure 5A. The next waypoint is the start point of the second loop $p_{0}^{2}$ . This process continues until all waypoints have been assembled into the list. The waypoint list is then published using the standard ROS message (*geometry-msgs/PoseArray.msg* ^11^) and the robot is ready to begin the process of navigating to the waypoints in turn.

#### 4.3.2 State machine

**FIGURE 6:**
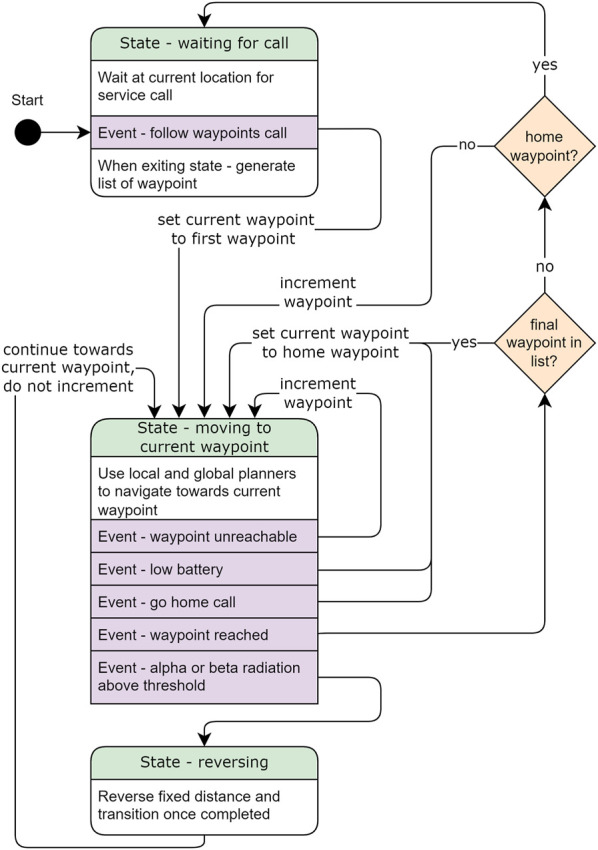
Flow diagram showing the operation of the state machine that controls the navigation of CARMA II. The three states are represented by the rounded boxes with state names highlighted in green, events that trigger transitions are highlighted in purple, transitions are represented by the black arrows and decisions during transition are represented by the diamonds.

The *state-machine* node was written to implement the custom behaviours required for CARMA II such as reverse motion and re-planning on detection of alpha or beta contamination, return home when battery is low and the ability to re-plan if waypoints are unreachable. The flowchart of the state machine is presented in Figure 6.

When CARMA II is started up it enters the *waiting for call* state, where CARMA II holds its current position. While in this state the operator uses the waypoint planner’s graphical interface to plot the coverage area of CARMA II. To begin the survey, a ROS service call is used to trigger the *service call* event. This event causes the *waiting for call* state to publish the list of waypoints, sets the current waypoint to first waypoint in the published list, and then transitions to the *moving to current waypoint* state.

In the *moving to current waypoint* state, CARMA II uses the costmaps combined with global and local planners to control motion of the robot toward the current waypoint while avoiding obstacles and regions with high radiation levels. While in this state, CARMA II continuously monitors for the events: *Waypoint unreachable*, *low battery*, *waypoint reached*, *alpha or beta radiation above threshold*. If any of these events occur, CARMA II exits this state and enters a transition, as detailed in Figure 6. In the case that the current waypoint is not reachable, for example, if the waypoint is placed inside an obstacle or a newly discovered radiation patch, the next waypoint will be sent as a goal location for path planning and navigation. If battery drops below a preset threshold, or the user triggers the *go home call* event the robot briefly leaves the *moving to current waypoint* state and changes the current waypoint to the home location before re-entering the state. If waypoint is reached this triggers two decisions. If CARMA II has reached the final waypoint in the goal list, the current waypoint is updated to be the home location. If the waypoint that has been met is the home waypoint, CARMA II transitions to the *waiting for call* state.

When the RadEye SX reports levels of alpha or beta radiation that is above a given threshold, the *alpha or beta radiation above threshold* event is triggered and the robot transitions to the *reversing* state. In this state, CARMA II will reverse a fixed distance and the radiation costmap will be automatically updated. The local planner then modifies the path of the robot to reach the current waypoint while avoiding the area that was found to have high levels of alpha or beta radiation. The reversing distance is a function of the forward velocity of the robot, the rate of the reporting sensor and the robot’s maneuverability. To avoid the wheels becoming contaminated, the forward velocity of the robot (*v* m/s) is a function of the distance between the robot’s wheels and the probe (*d* m), and the refresh interval of the sensor (*t* s): *v* < *d*/*t* m/s, For a RadEye SX with an update rate of 1 Hz and forward velocity of 0.2 m/s, the robot should reverse at least twice the distance covered per sensor reading (0.4 m) to avoid re-triggering the sensor. To include a sensible safety factor and allow the robot to more easily plan and manoeuvre around the new radiation patch, this is minimum distance is increased to 1.0 m in this study. When the reversing motion is completed CARMA II exits the *reversing* state and transitions to the *moving to waypoint* state. It is important to note that the current waypont is not incremented after reversing. If the current waypoint has become blocked by radiation, the *waypoint unreachable* event will be triggered as soon as the robot re-enters the *moving to waypoint* state and the current waypoint will then be incremented.

Avoidance of gamma radiation has no associated states in the state machine and is implemented *via* direct interaction with the radiation costmap. It was deemed unnecessary to have special motions, such as the reversing motion, for gamma radiation. This is because, due to its high penetration, gamma radiation can be easily detected and does not carry the same contamination risks as alpha and beta. Moreover, the method used has already been proven to be effective in a previous publication by the authors (West et al., 2022).

The *state-machine* node was implemented using the SMACH package and was integrated with the ROS navigation stack: *Move-base-flex*. In addition to the go home service call, the monitoring process can be hard stopped by an operator using the emergency stop button on the top of CARMA II or the wireless emergency stop button next to the base-station.

## 5 Physical evaluation and discussion

To assess the performance of the CARMA II and its state machine in avoiding spread of alpha contamination, as well as its compatibility with gamma radiation avoidance, a set of experiments was conducted. Simulated alpha and gamma radiation sources were used, the positions of which were known to the authors, but unknown to the robot during each experiment. As alpha and beta contamination have a short path length in air, the robot can only observe this when the detector is in close proximity of radioactive material. For gamma radiation, observed intensity follows an inverse square relationship with distance, i.e., being very close to a source leads to dramatically higher observed intensity, therefore, any increase in distance from a source will yield diminished accumulated dose.

For each run, the robot was instructed to perform a coverage path consisting of four concentric polygons, with the inner most polygon being the first to be completed and the outer being the last to be completed, with a connecting edge between the polygons at one vertex as discussed previously in Section 4.3.1. The start of the coverage is aligned with (0,0) in each figure.

### 5.1 Detection and avoidance of alpha radiation

**FIGURE 7:**
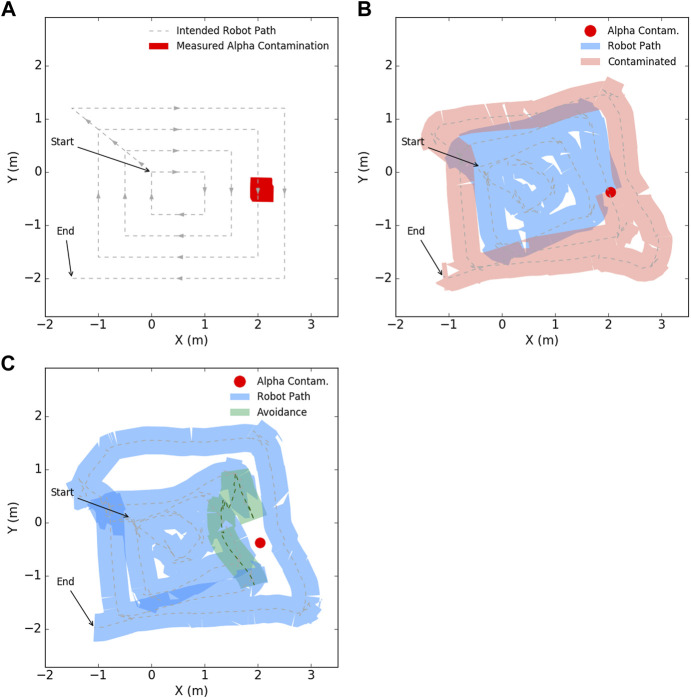
A four loop coverage path in the presence of measurable alpha contamination. **(A)** shows the generated path of the robot with estimated location of alpha contamination, **(B)** shows the actual path of the robot with possible spread of mobile contamination, with the width of the robot chassis indicated by the thickness of the path, **(C)** shows the robot reversing and maneuvering around the observed contamination due to the state machine logic in this work.

In the first experiment, the ability of the robot to detect and avoid exposure to alpha contamination was investigated, as mediated by the developed state machine. As the robot utilises localisation, the position of any measured alpha contamination can be referenced to a specific coordinate for end users, as shown in Figure 7A. The rectangular footprint of the contamination estimate is due to the dimensions of the detector itself.

Figure 7B shows the path the robot takes whilst carrying out its autonomous navigation, with the width of the coloured trace corresponding to the width of the robot. When the robot passes over the region of alpha contamination the change in color represents possible spread of mobile contamination due to its contact with the wheels. As is demonstrated, such undesirable spread of contamination could be considerable depending on the conditions of the radiation material and the environment.

When the robot encounters an increase in alpha radiation intensity above a threshold, the previously described state machine (Section 4.3.2) successfully enacts an autonomous reverse motion and plans a path which navigates around the suspected area to continue its coverage. In Figure 7C, this is demonstrated by the path of the robot not intersecting with the location of measured alpha radiation (red region), with the reverse and avoidance highlighted in green.

It is observed in Figure 7C that CARMA II does not always track a straight line between waypoints. This can be due to the costmaps generated by radiation detectors or obstacle detection, but is most often related to the behaviour of the local trajectory planner. As the robot reaches each waypoint, it does not adjust its heading to point towards the next waypoint before moving forwards. Rather the robot begins to move and adjusts it’s heading while progressing forwards. This can cause apparent skewing of the actual path relative to the straight line path and can in some cases cause some deviation as can be seen on the top left side ofFigure 7C.

### 5.2 Detection and avoidance of gamma radiation

**FIGURE 8:**
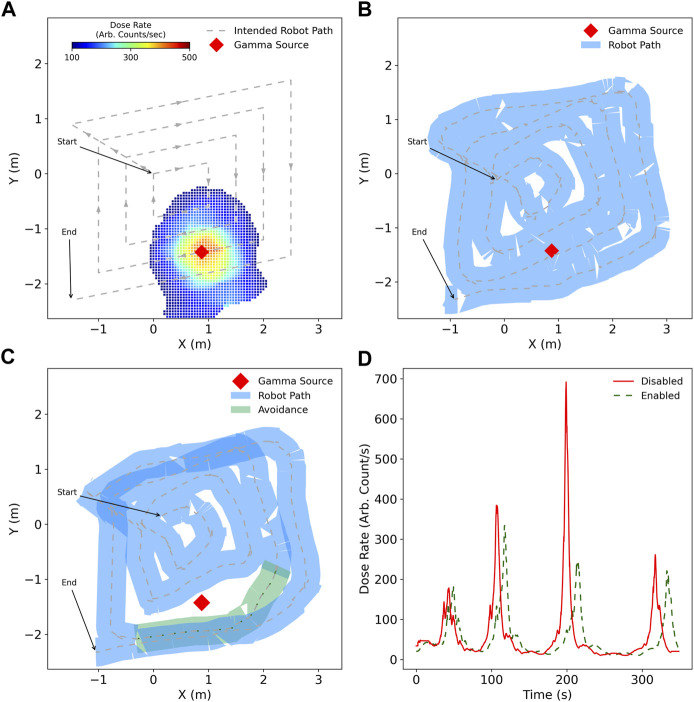
Coverage survey of four loops with no alpha radiation present but measurable gamma. **(A)** generated coverage path with location of gamma point source along path along with the interpolated gamma radiation field estimated by the robot, **(B)** actual robot path with no situational awareness of gamma radiation which brings it into close proximity, **(C)** the robot plans a path around the gamma source to decrease unnecessary exposure, **(D)** the difference in gamma radiation exposure during the entire autonomous survey.

In this experiment, the extent of coverage was investigated with the functionality of gamma radiation avoidance enabled. A mock gamma radiation point source was placed in the vicinity of *L*_3_ of the robot coverage pattern as shown in Figure 8A. The robot performed an autonomous survey for alpha contamination whilst also monitoring gamma field intensity. Figure 8A also shows the map of gamma radiation generated by the robot as it performs its inspection, whilst also having radiation avoidance enabled. This map could be used by stakeholders to plan future human or robot inspection tasks.

With additional situational awareness, the robot was capable of reducing its total accumulated gamma exposure by 13% whilst maintaining its coverage plan for the majority of the survey. This reduction in exposure can be increased or decreased based on the user-specified thresholds, with the compromise of more complete or incomplete coverage, respectively. Figure 8D shows how gamma dose rate is significantly diminished around *L*_3_ when the robot has avoidance enabled compared to when avoidance is not enabled. For loops *L*_2_ and *L*_4_, there is also reduced exposure at the peak of the expected dose, demonstrating the compromise the robot takes to maintain the coverage path as a priority. Figures 8B, C show how the paths for loops *L*_2_ and *L*_4_ are very similar, exhibiting only small displacement away from the gamma source, whereas for *L*_3_ the robot takes more significant evasive action.

### 5.3 Simultaneous detection and avoidance of alpha and gamma radiation

The performance of CARMA II was finally investigated in a mixed radiation field of both alpha contamination and a gamma point source, to assess the robot’s capability to manage both evasion schemes during the same survey mission.

**FIGURE 9:**
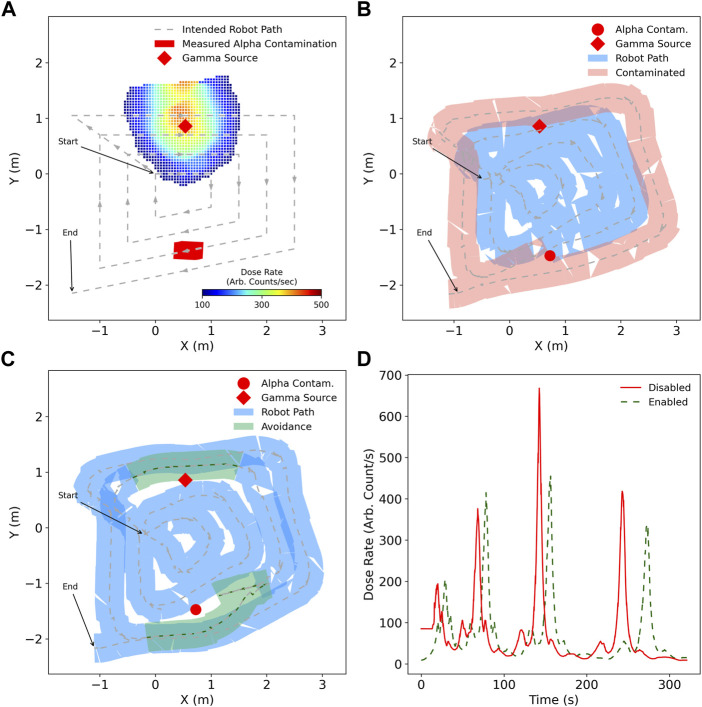
Survey of an environment utilising a four loop coverage path, with alpha and gamma radiation sources present. **(A)** the intended coverage path, with the measured radiation intensity by the end of the survey with avoidance enabled for alpha and gamma, **(B)** path taken by robot in blue when not aware of ionising radiation risks, with possible spread of mobile alpha contamination as a consequence, **(C)** robot path with broader alterations to the coverage path in green as a result of radiation awareness, **(D)** dose rate measured by the robot during the surveys.

Figure 9B shows the robot autonomously following the path generated in Figure 9A. The robot takes no additional action to avoid either the alpha contamination nor the gamma point source, with the possible contamination spread highlighted in light red as the footprint of the robot chassis passes over the contamination point.

In contrast, Figure 9C shows avoidance of both alpha and gamma sources by the *state-machine* and additional path planning cost, respectively. The alpha contamination is correctly avoided by performing a reverse motion and maintaining a minimum distance from the measured contamination location. For gamma avoidance, the path taken by the robot is modified to be further away from the source with respect to the default case when it is in very close proximity.

As was the case in Experiment 2, in Figure 9D the peak dose rate associated with the robot in close proximity to the gamma source is decreased for the avoiding case, with total accumulated dose being decreased as a result. As additional path planning cost is linearly proportional to dose rate, it is important to note that only when the dose rate is above a certain threshold, based on numerous factors, does the robot take any considerable action West et al. (2022). This ensures that coverage is prioritised, with only the significant threat to the health of the robot leading to a safer route.

### 5.4 Discussion

The experiments that were completed show that the developed robotic system can autonomously detect different types of radiation in hazardous nuclear environments and, more importantly, is able to respond to different types of radiation that are encountered. By manoeuvring away from and around mobile alpha contamination, the environment is protected from further radioactive material spread, and by keeping an increased distance from harmful gamma radiation, the robot can protect itself from possible damage to circuitry and materials. This avoidance behaviour forms part of the autonomous inspection that CARMA II is capable of.

Avoidance of gamma radiation sources create a beneficial deviation in the coverage path, whilst not introducing unnecessary alteration which may compromise coverage. This response can be tuned *via* costing thresholds in the path planner. As gamma radiation intensity follows an inverse distance square relationship, increased distance from a source is greatly beneficial when in very close proximity. The reduction in accumulated dose and appropriate costing thresholds must be made as a cost benefit analysis by stakeholders, based on robot radiation tolerance and return on investment metrics. The more cautious, the less radiation damage will occur at the expense of incomplete coverage.

Once a preliminary survey of an unknown or dynamic environment is made, future inspection missions can be tailored to assess the situation in more detail, rather than provide a broad overview. This can fill in any gaps in data collection due to avoidance, and help refine estimates of possible radiation locations. As shown in Figures 7A, 9A, the extent of alpha contamination is convolved with the dimensions of the paddle detector, and a secondary phase assessment process needs to be developed to ascertain the true extent of any contamination.

By including radiation avoidance, more specifically alpha and beta avoidance, a limitation to the speed of inspection is introduced. Radiation sensing typically has a low update rate in the order of 1 Hz, therefore, between a confirmed detection of highly localised alpha and beta contamination and this being reported to the robot, 1 s may have elapsed. Though the alpha and beta detection instrumentation is deliberately placed ahead of the robot chassis, this only affords the robot a short window of prediction on future conditions. The slower the robot, the more time it has to react, however, this is a waste of other resources such as robot battery capacity. Assuming that the sensor update period is *t* seconds, and the forward distance of the sensor is *d* metres, then to avoid contamination of wheels and other ground contacting features, the robot must limit its linear velocity, *v* < *d*/*t* m/s, to ensure, that with infrequent sensor updates, the robot cannot traverse too much area as this would increase the risk of contact with contamination. For CARMA II the sensors were placed approximately 0.25 m ahead of the robot chassis, with a publish rate of 1 Hz for the sensor. Therefore, CARMA II is limited to ≤ 0.2 m/s with this sensor configuration.

CARMA II has demonstrated that it can undertake monotonous room scale inspection tasks, generating a map of possible alpha and gamma contamination for health physics surveyors. Once possible radioactive materials have been identified, humans can act accordingly and plan to perform more specific and targeted inspection based on maps produced by CARMA II. Furthermore, any gaps in coverage which arise are completely known to end-users and can be addressed with subsequent robot or human inspection tasks. Use of radiation aware robotic systems such as CARMA II can greatly increase the productivity of health physics surveyors.

## 6 Deployments and future plan

To gain feedback from end users, an early prototype version of CARMA II was deployed at the Sellafield site in an arena that had known regions of alpha contamination. The main aim of deployment was to test CARMA II’s software systems and its radiation detection abilities in a real environment using real radiation sources. CARMA II successfully performed the first ever autonomous radiometric survey on the Sellafield site, during which it identified a known source of alpha contamination and placed this in the correct position on the map that it generated. Following feedback from end users, CARMA underwent a full hardware redesign to make the robot more robust, facilitate fast battery swapping and allow the DP8 probe to have adjustable height.

The new version of CARMA II, as presented in this paper, is now ready for testing on the Sellafield site, with the first deployments planned for early 2023. During the deployment period there will be continual feedback and improvements made to the robot. The intention is that CARMA II will be part of “business as usual” for Sellafield in the coming years and it will be a standard tool that is available to aid Health Physics personnel.

## 7 Conclusion

This article has provided the necessary background research to facilitate the use of autonomous ground vehicles for surveying floor areas in nuclear facilities for alpha, beta and gamma contamination. Unlike previous robots, CARMA II has the ability to map and avoid alpha, beta and gamma radiation simultaneously. Although this is a novel feature, the true benefit of CARMA II is its ability to detect alpha and beta contamination and subsequently re-plan its path to avoid the contaminated area. This reduces the risk of spreading contaminated radioactive material during the survey, which until now has been a key differentiating feature of using human operators over robotic systems. The developed system’s functionality and ability to simultaneously survey for, and avoid alpha, beta and gamma radiation was demonstrated in a series of experiments using a physical robot within a representative environment.

## Data Availability

The datasets presented in this study can be found in the online Figshare repositories https://doi.org/10.48420/21864987.v1 and the code developed is available in the GitHub repository, available on request.

## Footnotes

1
http://wiki.ros.org/slam_toolbox.

2
http://wiki.ros.org/move_base_flex.

3
http://wiki.ros.org/dynamic_reconfigure.

4
http://wiki.ros.org/pointcloud_to_laserscan.

5
http://wiki.ros.org/velodyne_pointcloud.

6
http://wiki.ros.org/ira_laser_tools.

7
https://github.com/EEEManchester/radeye.

8
https://github.com/EEEManchester/radiation_msgs.

9
https://github.com/EEEManchester/radiation_layer.

10
https://github.com/EEEManchester/waypoint_planner.

11
http://wiki.ros.org/geometry_msgs.
