# Corrigendum: CARMA II: a ground vehicle for autonomous surveying of alpha, beta and gamma radiation

**DOI:** 10.3389/frobt.2023.1237449

**Published:** 2023-07-05

**Authors:** Bahman Nouri Rahmat Abadi, Andrew West, Harriet Peel, Matthew Nancekievill, Christopher Ballard, Barry Lennox, Ognjen Marjanovic, Keir Groves

**Affiliations:** ^1^ Manchester Centre for Robotics and AI, University of Manchester, Manchester, United Kingdom; ^2^ Sellafield Ltd., Carlisle, United Kingdom

**Keywords:** autonomous ground vehicle, ROS, radiation detection, costmap, state machine, experimental

In the published article, there was an error in the **Author** list, and author “Harriet Peel” was erroneously excluded. The corrected **Author** list appears below.

“Bahman Nouri Rahmat Abadi^1^*, Andrew West^1^, Harriet Peel^1^, Matthew Nancekievill^1^, Christopher Ballard^2^, Barry Lennox^1^, Ognjen Marjanovic^1^ and Keir Groves^1^.”

The authors apologize for this error and state that this does not change the scientific conclusions of the article in any way. The original article has been updated.

